# The microbial ferrous wheel: iron cycling in terrestrial, freshwater, and marine environments

**DOI:** 10.3389/fmicb.2012.00383

**Published:** 2012-10-31

**Authors:** David Emerson, Eric Roden, Benjamin S. Twining

**Affiliations:** ^1^Bigelow Laboratory for Ocean SciencesEast Boothbay, ME, USA; ^2^Department of Geoscience, University of WisconsinMadison, WI, USA

Were Oscar Wilde a devotee of iron biogeochemistry in the twenty-first century (hard as that might be to imagine), he might remark that iron is the most ironic of elements. Those interested in microbes that carry out life-sustaining iron-coupled redox reactions like to point out that iron is, after oxygen, the most abundant redox-active element in the Earth's crust. However, those studying oceanic phytoplankton regard iron as a nutrient that occurs at such vanishingly low concentrations in the surface ocean that it limits the growth of algae in more than 40% of the global ocean. This is because at the pH of seawater oxygen promotes the rapid oxidation of soluble ferrous iron to insoluble ferric iron oxyhydroxides that precipitate and sink out of the water column. As a result, while many marine microbes, and especially the photosynthetic ones, have developed finely-tuned mechanisms for acquiring iron, total primary productivity can be limited by iron.

At oceanic hydrothermal vents, and in terrestrial habitats, iron is not a limiting nutrient. At many oxic-anoxic interfacial habitats, not only is iron not limiting, but it is so abundant that lithotrophic microbes can use it as an electron source to sustain growth, and form robust communities of iron-oxidizing chemolithoautotrophs. In more acidic conditions, such as certain hot springs and acid mine drainage systems, ferrous iron is more stable, concentrations can be in the millmolar range, and specific communities of archaea and bacteria that use iron as an energy source can flourish. Iron-oxidizing microbes are not limited to aerobic habitats, but can also oxidize iron under anaerobic conditions by coupling the oxidation to either anoxygenic photosynthesis or nitrate reduction. Nor does it appear that they are limited to only utilizing soluble ferrous iron as an energy source, but can also acquire iron from insoluble minerals that contain reduced iron.

But iron-oxidation is only one-half of the equation. The utilization of ferric iron, principally in the form of Fe-oxides, to carry out anaerobic respiration is well established as an important pathway for organic carbon metabolism in anaerobic habitats. Furthermore, model organisms such as *Shewanella* and *Geobacter* are utilized to study the biochemical mechanisms of Fe-reduction, and from this we have learned a good deal about processes involved in extracellular electron transfer. Taken as a whole, it is apparent that the iron cycle is a remarkably complex process, dependent upon a wide range of chemical interactions, habitat types and groups of microbes that link it to all of Earth's other important biogeochemical cycles.

In this special topic issue we have gathered contributions from scientists working in diverse disciplines who have common interests in iron cycling at the process level and at the organismal level, from the perspective of iron as an energy source or as a limiting nutrient for primary productivity in the ocean. The hope is that bringing together seemingly disparate lines of research under one cover will result in a more global understanding of the iron cycle, and perhaps draw new insight into the connections within the cycle. We were very fortunate to enlist a varied and talented group of authors to contribute a wide range of articles. In total, 16 papers have been included, with a mixture of 9 original research articles, 6 reviews, and 1 perspective.

Aspects of iron cycling in the open ocean are covered by reviews on organic complexation (Gledhill and Buck, 2012) and on the role of superoxide dismutase (Rose, 2012), as well as in a research article on the role of weak iron-binding ligands in the ocean by Croot and Heller (2012). Oxygen-dependent iron oxidation at circumneutral pH is addressed in a research paper on a potential mechanism for iron oxidation by Liu et al. (2012), a research paper on mineralogy of biogenically-formed oxides at a hydrothermal vent (Toner et al., 2012), and a review of iron-based ecosystems associated with hydrothermal vents and the subsurface in the Pacific by Kato et al. (2012). Iron-cycling in acidic systems is reviewed by Johnson et al. (2012), and original research on a unique iron-rich acidic ecosystem in Yellowstone National Park is presented by Kozubal et al. (2012). A novel spectroscopic technique for biochemical analysis of iron oxidation in *Leptospirillum ferroxidans* is contributed by Blake and Griff (2012). Microbial utilization of iron under anaerobic conditions is dealt with in a review of mechanisms for iron reduction by Shi et al. (2012). Picardal (2012) reviews abiotic and microbial interactions of anaerobic iron oxidation and Carlson et al. (2012) provide an interesting perspective piece on nitrate-dependent iron oxidation. Original research on iron reduction in *Shewanella* is presented by Coursolle and Gralnick (2012), and competition among phototrophic and nitrate-dependent iron-oxidizing microbes is addressed by Melton et al. (2012). Finally, original work from the laboratory of Eric Roden investigates redox cycling in a typical freshwater iron-rich stream (Roden et al., 2012), as well as the capacity for microbes to use iron minerals as an iron source (Shelobolina et al., 2012).

Taken together these papers present an overview of research on iron from a range of perspectives that indicates the breadth of work that has been done and provides insight into the many exciting avenues of research that continue to enhance our understanding of the iron cycle in nature.
